# A Two-Step Sensor Fusion Methodology to Assess Damage on Drone Propellers by Audio and Radar Measurements

**DOI:** 10.3390/s26051429

**Published:** 2026-02-25

**Authors:** Gianluca Ciattaglia, Giacomo Peruzzi, Matteo Bertocco, Valeria Bruschi, Stefania Cecchi, Grazia Iadarola, Alessandro Pozzebon, Susanna Spinsante

**Affiliations:** 1 Dipartimento di Ingegneria dell’Informazione, Università Politecnica delle Marche, 60131 Ancona, Italy; g.ciattaglia@staff.univpm.it (G.C.); v.bruschi@staff.univpm.it (V.B.); s.cecchi@staff.univpm.it (S.C.); g.iadarola@staff.univpm.it (G.I.); s.spinsante@staff.univpm.it (S.S.); 2 Dipartimento di Ingegneria dell’Informazione, Università degli Studi di Padova, 35131 Padova, Italy; matteo.bertocco@unipd.it (M.B.); alessandro.pozzebon@unipd.it (A.P.)

**Keywords:** unmanned aerial vehicle, fault detection, embedded machine learning, audio signals, MEMS, microcontroller, radar FMCW, vibrations

## Abstract

Safety in the operation of Unmanned Aerial Vehicles (UAVs) is emerging as an increasingly important requirement to avoid accidents or possible hazards, because of the growing number and variety of applications that make use of such systems. Consequently, the ability to detect and classify damages occurring on UAV components becomes critical, so that appropriate countermeasures can be applied on time. In this paper, a two-step methodology is proposed to detect damage to UAV propellers, and to classify its severity, so that the most appropriate response can be implemented. In fact, a first step is carried out onboard drone, in real-time, taking advantage of the acoustic emissions of the propeller and the potential of edge processing: a tiny Machine Learning (ML) classifier assesses the severity of the damage and, when deemed critical, the UAV is directed towards a ground station hosting a radar-based system, to discriminate the severity of the fault based on contactless vibration displacement and frequency measurements. The combination of both detection approaches realizes a diagnostic system that is time-responsive and accurate in defining the type, the amount, and the location of the damage. Damage classification performance values over 99% are provided by the embedded audio-based ML model; the radar-based step can further differentiate and measure the location of the propeller cut, which could eventually lead to forced landing of the UAV.

## 1. Introduction

The adoption of Unmanned Aerial Vehicles (UAVs) has become widespread in different contexts, not only limited to the industrial domain, where they can be equipped with various sensors, like vision, thermal, and acoustic ones, to monitor the conditions of machinery and infrastructures [1]. UAVs are used in many scenarios, far beyond recreational purposes, spanning from military to civil and post-disaster applications [2,3] and, in the future, in smart cities [4,5,6], where they have been increasingly integrated into initiatives across various domains, including safety [7], security, surveillance, disaster management, emergency response, and transportation. The literature emphasizes the importance of addressing not only security and privacy concerns related to drones, by leveraging Internet of Things (IoT) and Artificial Intelligence (AI) technologies, but also safety issues and advanced control methods to maximize the benefits of UAVs in smart cities, while reducing the probability of dangerous consequences related to their use [8,9].

To push this technology further and use it massively, a very high level of safety must be achieved, similar to other commercial aerospace technologies. Such a level of safety can be reached by ensuring that any fault occurrence is early detected, and a countermeasure is applied on time. Military drones ensure an extremely high level of fault tolerance; however, this is achieved at the expense of an amount of complexity and costs which are not sustainable in civil UAVs, which need to be cheaper. To be viable, any solution aiming at increasing the safety levels of these systems must be implemented taking into account limits on bearable power consumption and costs. In response to these two needs, it is possible to envise a multi-step strategy to detect drone malfunctions and implement an efficient and effective maintenance approach, with a twofold aim, namely to minimize the risk of accidents and related consequences, and to avoid the disruption of the service relying on the use of the drone itself, by implementing a contactless diagnostic of the UAV. A first check of the drone health status may be performed onboard, by means of lightweight sensor technologies and signal processing techniques applied to fault detection. Then, as soon as a potential fault is detected, a second deeper check may be performed by resorting to more resource-demanding equipment, located at fixed ground positions to which the UAVs may be driven by a proper supervising system.

In this context, the reduced cost and the increased performance of microcontrollers and embedded systems are acting as enablers for a large number of solutions once demanding for high performance computing tools. This is the case of Machine Learning (ML) algorithms which can nowadays be trained to be then employed for classification purposes directly on the edge. Together with the complexity of the algorithms to be implemented, another factor that has to be taken into account when designing any embedded system is related to the source of signals used as inputs for the ML algorithms themselves: indeed, the integration of signal sources like cameras poses great constraints for what concerns the overall energy requirements of the system. For this reason, the signals to be analysed should come from low-complexity sources, whose contribution to the overall energy budget of the system itself should not harm its correct functioning.

Various activities have been explored concerning ML techniques applied to drones, ranging from drone detection in anti-intrusion systems [10,11] to the identification of their direction, payload, and potential malfunctions. Detection of harmful conditions also involves signals such as vibrations, images, radar [12], and audio signals, with a focus on utilizing sounds emitted by drone propellers [13]. The application of ML or Deep Learning (DL) is common in designing sound-based drone detectors [10,14], where extracted audio features play a key role in distinguishing drones from acoustically similar objects.

Regarding fault detection, in [15], a prototype of a diagnostic system intended to recognize and identify broken blades of rotary wing UAVs is presented. The solution is based on an analysis of acoustic emissions recorded with an onboard microphone array paired with a single-board computer. The detection of UAV motor faults by acoustic processing is addressed in [16], testing three different classifiers on three different UAV motors. In [17], a multitask learning model is proposed, based on a deep neural network, to identify faults in propellers and motors. The audio features are extracted from operating sounds of drones collected from microphones mounted on three different drones, in an anechoic chamber, as was done in this paper.

Most of the ML- and DL-based approaches are designed and validated on high-power processing units. Very few contributions tackle the problem from an embedded ML point of view. Additionally, the majority of available works exploits collections of sound files that have been recorded under different conditions, often without providing details about the sound measurement chain or the equipment used. Often, Micro Electro-Mechanical Systems (MEMS) microphones are used in the studies, without any calibration or characterisation procedure.

**Figure 1:**
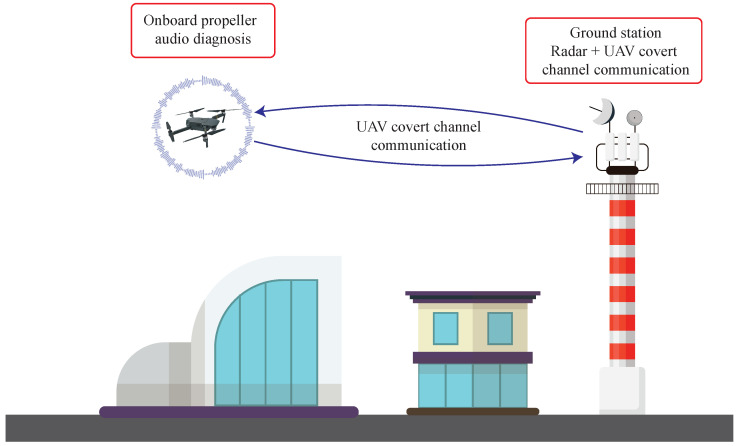
Graphical representation of a possible smart city scenario in which drones support a variety of services and their safety is monitored by the proposed two-step approach.

In the specific context highlighted above, and taking into account the last considerations, this paper extends the previous work [7] to validate the feasibility of a system capable of detecting damages on quadcopter UAV propellers. The proposed approach is grounded on two steps: the former is based on an onboard embedded ML audio system able to roughly classify the type of probable damage affecting a propeller, while the latter is based on a ground-based radar sensor able to provide a more detailed quantification of the damage by contactless measurements. In so doing, a fault detection paradigm relying on a non-contact sensor fusion technique is carried out. While ML has already been exploited in the literature for the classification of drone faults through audio signals, the objective of this paper is to demonstrate the viability of such a solution in a condition of limited hardware resources. Both methods are complementary and compensate for each other shortcomings. The audio method can be applied in real-time, on board the UAV, providing alert information, while the radar one can better detail the type of damage. For this purpose, a specific setup is designed for both data acquisition and processing, taking into account the final deployment of the system in a possible scenario like the one shown in Figure 1. This work pays attention to the proper measurement and collection of audio signals by means of both professional laboratory equipment in a semi-anechoic chamber, avoiding the influence of environmental and external noise, and an embedded acoustic measurements acquisition setup, installed onboard the drone. This gives rise to several advantages. First of all, the collected dataset exploiting the professional setup gives the possibility of simulating different scenarios, by adding other noise types (such as road traffic or wind noise) to the clean measurements acquired in the chamber, thus paving the way for further different experiments. Secondly, in the case of sound recordings from a drone in operational conditions, it is possible to apply a denoising algorithm to isolate the contribution of the drone from the environment, and make a direct comparison with the proposed dataset. Then, the exploitation of the embedded measurement system allows for the acquisition of acoustic data directly onboard the drone, accurately reproducing real operational working conditions.

While it is well known how radar systems can measure a target range distance, velocity, and Angle of Arrival (AoA), less attention is paid to their capability to measure the target vibration displacement. This possibility is provided by the micro-Doppler effect, a phenomenon that can be exploited to measure the target vibration which can be used also to identify the drone [18]. In particular, the radar can detect the position of the target, or of a specific part of it, and extract the micro-Doppler information, from which the vibration displacement signal can be retrieved. Therefore, the application of this technique to a specific part of the UAV chassis enables the method proposed in this work. Although the use of the micro-Doppler effect for classification purposes is quite common, this is not able to provide useful quantities for recognizing a possible fault. However, by applying a method that converts this phenomenon into a displacement measurement, it is possible to obtain quantities that can be used to diagnose faults without resorting to computationally burdensome techniques [19,20].

The paper is organised as follows: Section 2 details the envisioned application scenario of UAVs in smart cities and motivates the proposed multi-stage approach to fault detection and predictive maintenance. Section 3 describes the experimental setup used to perform audio signal acquisition from the drone, by applying a controlled and repeatable damage to its propellers; the same section also shows the experimental setup for radar signals collection. Section 4 details the acquired datasets of audio and radar signals, while Section 5 describes the ML model used in this work. Results are presented and discussed in Section 6, and finally Section 7 concludes the paper.

## 2. Application Scenario

The developed approach finds room within a Smart City application scenario, in which UAVs are exploited for sundry purposes: from patrolling to monitoring, from logistics to deliveries, from urban traffic monitoring to rescue procedures, and so on (as graphically shown in Figure 1). Because of such a context, ensuring safety related to UAVs while they are flying is of utmost importance, in order to avoid (or at least to minimize) dangerous outcomes (e.g., consequences to things or people, hindrances of infrastructure operations, injuries, or even casualties) resulting from unexpected crashes caused by propellers damage. Therefore, setting up a continuous damage detection strategy for drone propellers, guaranteeing an effective and accurate predictive maintenance policy while not disrupting the provided service, is pivotal. However, this implicitly poses a trade-off. According to the literature, predictive maintenance of drones can also be done without resorting to additional sensors, by exploiting Kalman filtering techniques on their motor control signals: such an approach is feasible just for a limited set of hovering flight modes, since a linearisation step of the nonlinear dynamic model is necessary. On the other hand, if supplementary and light onboard devices are included, then a coarse predictive maintenance can be continuously performed regardless of the route the UAV is following, while an accurate maintenance can be carried out by exploiting precise instruments that require to be hosted in ground stations for a plethora of reasons (e.g., mains powering, size and weight of the equipment, computational burden of the signal processing to perform, etc.).

Owing to this problem, we opt for devising a non-contact two-stage paradigm. The former takes place onboard the drone, which is equipped with an embedded device capable of continuously sampling the audio noise generated by the drone propellers. Such a device features an embedded ML model inferring propellers damage occurrences in real-time, and providing preliminary discrimination. This is a deliberate choice to keep the ML model as simple as possible so that it can be run by a microcontroller relying on a limited computational capability. Then, the model outputs can be promptly signalled to ground stations. There, the information retrieved by the onboard embedded ML model gets fused with that deriving from the radar measurements. Indeed, this work finds room within a broader framework, where drones are capable of being identified, as well as to communicate towards ground stations, by exploiting vibration fingerprinting techniques, thus setting up a covert channel between the drones and the radar-based ground stations. This was additionally proven in previous works. Specifically, [11] proved that radar-based ground stations are able to detect the type of propellers mounted on drones by means of non-contact measurements, while [21] proved that a covert channel, in which the drones can communicate by modulating their vibrational patterns, by accordingly modulating the control signals driving their propellers, can be set up towards ground stations. In light of this, by leveraging on such results, drones can communicate towards ground stations the inferences produced by the embedded ML model running onboard, to lately fuse them with the radar measurements.

The supposed ground station is placed in an environment where different radio technologies can be involved. Thus, the selection of the radar ensures compliance and compatibility with other communication systems. For this reason, the evaluation of the proposed method can be based on automotive radar sensors. In fact, they are designed to coexist in a Smart City context, with working frequencies spanning from 76 GHz to 81 GHz. Such a frequency band is split into two portions: the former from 76 GHz to 77 GHz is used for long range applications covering distances from 30 m to 300 m, and the latter from 77 GHz to 81 GHz for short range applications covering up to 30m[22,23]. As the development board used in this work is designed to fit these distances and to detect objects with bigger size than the used drone, the short-range distance scenario is exploited. Such a choice is motivated only by the characteristics of the commercial board used, but this limit can be overcome with a custom board design. The technical characteristics of the radar sensor must fit vibration measurement purposes. Automotive-grade radars are suited for the selected application scenario, considering their 120° field of view and the possibility to monitor multiple targets at the same time [24]. As the morphology of Smart Cities (or the environment where the system is employed) can vary, an accurate planning of the spots of radar ground stations must be provided, before installation in a real context environment.

Both methods work in synergy to promptly identify possible damage to the propeller. In a Smart City environment, the UAV cannot always be in a visibility region of the radar. By exploiting the audio noise signal, it is possible to obtain preliminary information about the damage to the propeller. Once the damage is detected, it is possible to exploit the radar capabilities to better understand the damage extent and make a decision about re-entering the UAV or leaving it to operate.

## 3. Experimental Setup

This section provides a description of the materials and equipment used to perform a series of tests aimed at evaluating the proposed approach. The setup used is composed of a custom drone, which is under test and placed over a metal tripod. In all experimental validation tests, a single assembled drone model is used. The reason is that, although the method may require a numerical tuning, by itself it is intrinsically independent of the drone model. The setup is arranged inside a semi-anechoic acoustic chamber where the audio acquisition system is connected to three microphones placed over separated stands. Then, the same chamber simultaneously hosts the acoustic measurements acquisitions performed with the embedded system, to improve the comparison between results, by minimizing confounding factors. The radar signal acquisition phase was carried out in the same environment as well. In Section 3.1, the drone setup is described, in Section 3.2 the audio acquisition systems are presented, while in Section 3.3 the radar acquisition system is shown.

### 3.1. Quadcopter Setup

**Figure 2:**
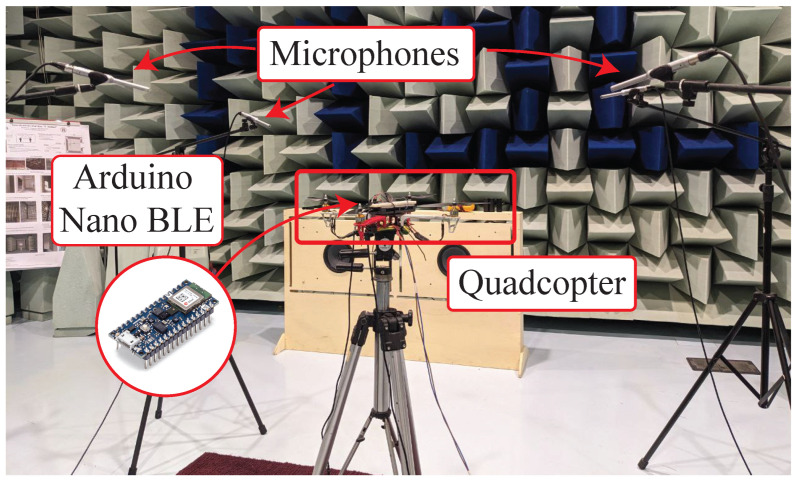
Experimental setup: in this photograph the quadcopter is placed over a tripod with three microphones around, the Arduino Nano 33 BLE Sense is installed at the centre of the drone chassis.

The UAV used for validating the proposed method is a customizable quadcopter, fixed onto a metal tripod. The DJI F450 four motor slots frame is used as a chassis, which allows us to modify the drone-related portion of the configuration. The materials of each frame are metal and plastic for the central section, and plastic for the arms, with a wheelbase of 450mmin length. The Brushless Direct Current (BLDC) motors feature $K_{v}=1000$ Revolutions Per Minute (RPM)/Vand are driven by four 40 A Electronic Speed Controller (ESC) drivers, supplied by the Matrix MPS-3005L-3 DC bench power supply unit. The latter provides 12Vvoltage to the power connector. The rotation speed of the BLDC motor can reach the maximum value of 12,000RPM with a $K_{v}$ of 1000RPM/Vand 12Vof supplied voltage. Each ESC unit is controlled by a Pulse Width Modulation (PWM) signal to set the rotation speed of the motors, featuring a frequency of 50Hzand a $T_{on}$ in the range from 1 ms to 2 ms. The resulting Duty Cycle ($D_{c}$) is in the range 5% to 10%; a $D_{c}$ bigger than 5% is needed to start the propeller rotation, while with $D_{c}$ = 10% it is possible to reach the maximum rotation speed. The PWM signal has an amplitude of 5Vand is generated by a National Instruments myRIO-1900 that drives the four motors with the same control signal [25]. The described setup is shown in Figure 2.

At each motor, one ABS plastic propeller is installed: the type used is composed of two blades with a total length of 254±1mm. The propeller model is 1045, where 10 indicates the diameter in inches, and 45 the slope which is 4.5 inches. The tests are performed in a semi-anechoic acoustic chamber, so that an open environment without any type of acoustic interference can be simulated. The quadcopter is positioned horizontally over the tripod to simulate the hovering flight condition.

### 3.2. Audio Acquisition Systems

#### 3.2.1. Data Acquisition System Using Professional Microphones

To detect and identify the type of fault on the propeller, the audio acquisition system must be equipped on the drone in an applied context. The required features depend on the desired performance. As different embedded microphone technologies provide different performance, and their evaluation is outside the scope of this work, professional microphones were chosen. They provide reference measurements, hence guaranteeing that the obtained performance is not affected by potential shortcomings or defects that may instead occur whenever embedded systems are at hand. One should note that the proposed setup means the method is based only on the sound emitted by the propellers, as it has been demonstrated that embedded microphones may directly collect information due to chassis vibrations (see [26,27,28], demonstrating how an embedded microphone can gather vibrations).

**Figure 3:**
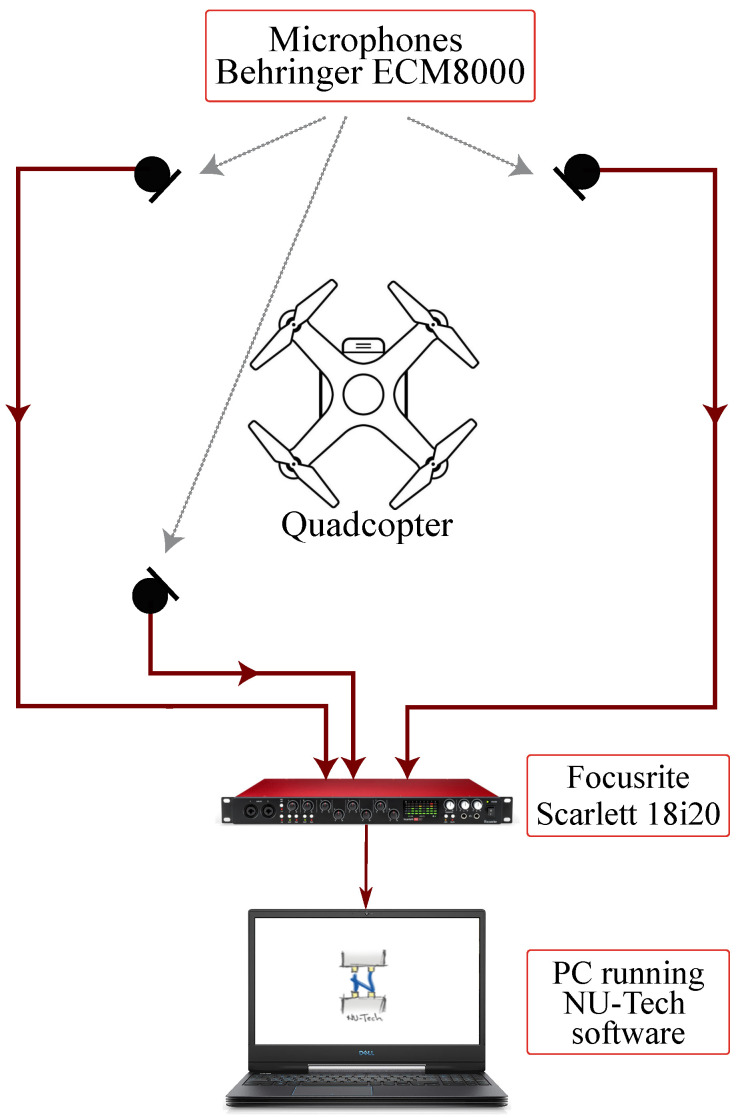
Audio acquisition system setup.

In detail, the audio acquisition system is composed of three professional XLR microphones (Behringer ECM8000) managed by an Asio sound card (Focusrite Scarlett 18i20) connected to a PC running NU-Tech software [29], as shown in Figure 3. The microphones are positioned around the quadcopter and they are arranged at three vertices of an ideal $1\;\times 1\;m^{2}$ square. As visible in Figure 2, all measurements are performed within a semi-anechoic chamber, ISO 3745 [30] certified, located at Università Politecnica delle Marche, Italy, to ensure a low environmental noise. Before the acquisitions, the microphones were calibrated using the Bruel&Kjaer 4231 calibrator and a digital Volume indicator (VUmeter) of the NU-Tech software [29]. Each signal lasts 116sand is saved in wave format (“.wav”).

#### 3.2.2. Embedded Data Acquisition System

As already noticed, the experimental setup includes an embedded acquisition system installed on the drone chassis. Namely, an Arduino Nano 33 BLE Sense, embedding a low-cost microcontroller (i.e., the Nordic Semiconductor nRF52840 32-bit ARM Cortex M4 running at 64MHz) was chosen. The board features an STMicroelectronics MP34DT05 omnidirectional digital microphone, which is a MEMS integrated sensor sampling audio at 16kHz. Moreover, the microcontroller provides a 256kBRAM, and 1MBof flash memory. Thanks to its small size and light weight, the board suits well in the drone, in view of real-time detection of propeller faults. As detailed in Figure 2, the board is installed in a central position with respect to the drone structure, and is mechanically integral to the drone chassis. To perform audio signals acquisition by the integrated MEMS microphone during the experiments, the board is connected via USB to a personal computer running a Python script, responsible for acquiring and storing the embedded acoustic measurements.

### 3.3. Radar System and Displacement Measurement Method

**Figure 4:**
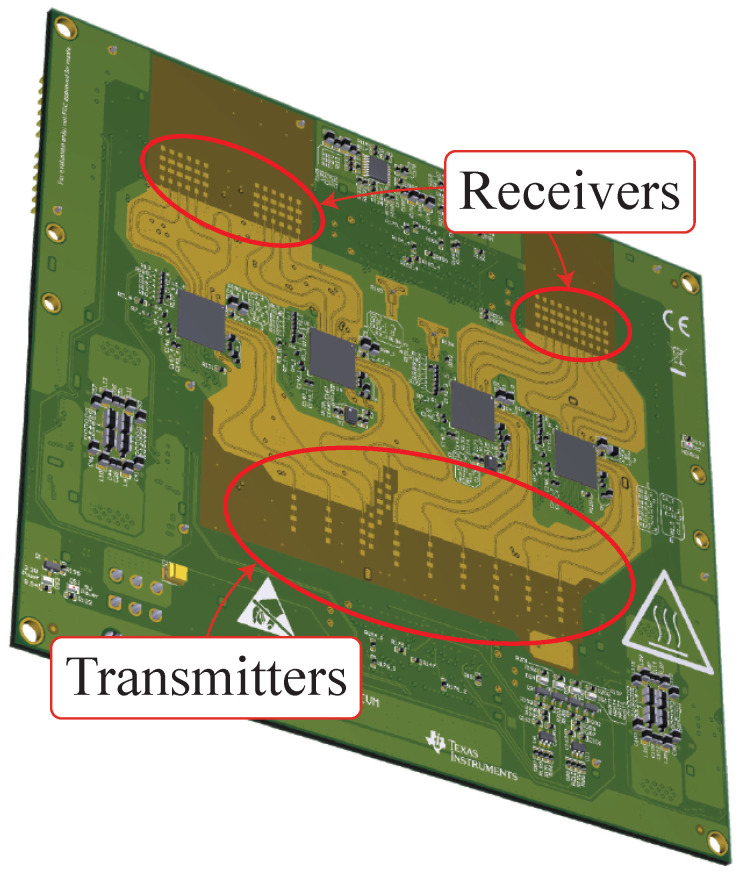
Texas Instruments cascade radar board [32]. The transmitter and the receiver arrays are highlighted with red circles.

**Figure 5:**
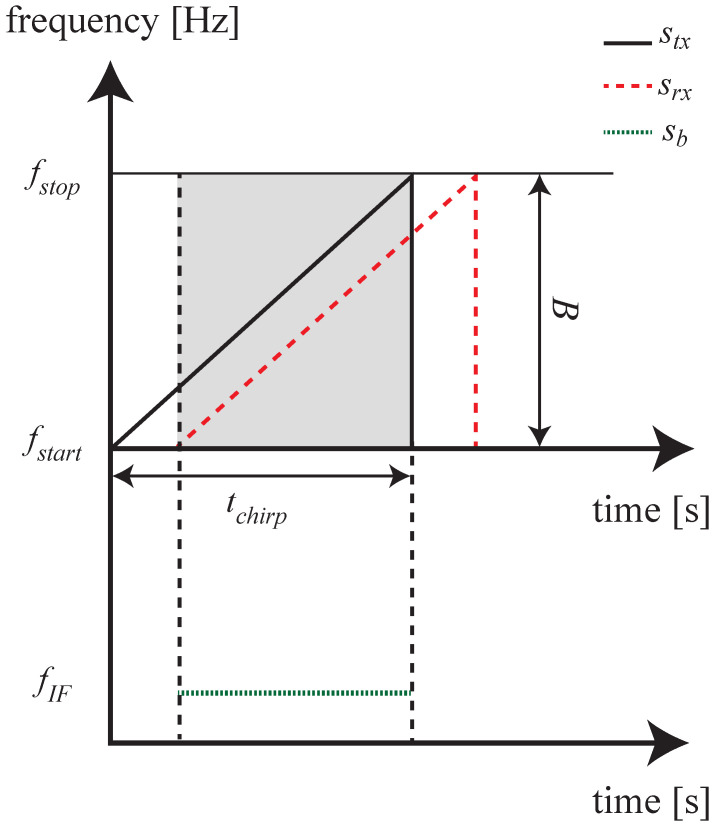
Chirp transmission ($s_{tx}$) and receiving ($s_{rx}$) scheme. Inside the grey overlap window, the mixing operation is performed. The obtained beat signal is indicated with $s_{b}$.

As mentioned in Section 2, the radar sensor used and shown in Figure 4 is developed for automotive applications. Automotive radars exploit a linear frequency modulated continuous wave (FMCW) modulation, so the transmitted waveform is a linearly modulated chirp. Unexpectedly, these particular types of radars do not transmit chirps continuously, since in between two consecutive chirps a waiting time is experienced due to the device implementation. The transmitted waveform will be only upchirp as the applied technique is slope independent. The Texas Instruments (TI) radar used is composed of four single chip radar sensors from the same company. The reason is the multiple-input multiple-output (MIMO) performance that can be achieved by combining the single chip MIMO capabilities. Indeed, a single chip features three transmitters and four receivers, and with the combination of all the chips it is possible to obtain a total of twelve transmitters and sixteen receivers on the same device. The result is a receiving virtual array of eighty-six elements along the azimuth plane. This value is the result of the transmitters and receivers collocation [31]. The number of virtual elements is fundamental for the target angular identification. This step is very important for the correct evaluation of the drone propeller vibration measurement [31], since with a field of view of 120° the angular resolution is 1.4° The FMCW modulation scheme requires the mixing operation between the transmitted and the received chirps. The operation produces an intermediate frequency signal called beat signal (i.e., $s_{b}$), which can be modeled as

(1) $$s_{b}\left(t\right)=s_{tx}\;\cdot \;s_{rx}^{*}\approx A\exp\left[j\left(2\pi \frac{B}{t_{chirp}}t_{d}t+2\pi f_{c}t_{d}\right)\right],$$

where *A* is the beat signal amplitude, $s_{tx}$ and $s_{rx}$ are the transmitted and the received chirps (the slopes of which are calculated as the bandwidth divided by the chirp duration, which is in turn indicated as $B/t_{chirp}$ and only takes positive values), the time *t* is defined as Fast-Time and ranges in the interval $\left(n-1\right)\;\cdot \;t_{chirp}<t<n\;\cdot \;t_{chirp}$ (where *n* indicates the *n*-th transmitted chirp, and $t_{chirp}$ is the chirp duration) and represents the time in which the signal is sampled by the Analog to Digital Converter (ADC), and $t_{d}$ is the delay between the transmitted and received chirps. The samples of the beat signals are collected in the overlapped timespan, as highlighted in grey in Figure 5.

The collected samples are stored on the radar hard drive and can be downloaded later to be processed offline. The method used to extract the vibration signal of the propeller under test is based on these samples. As the radar system is a development board, it is possible to configure many parameters of the transmitted waveform. This makes it possible to customize the application of the radar for setting up different parameter values from the ones typical of automotive scenarios. The configuration can be sent to the device through a dedicated software installed and running on the controlling computer.

**Figure 6:**
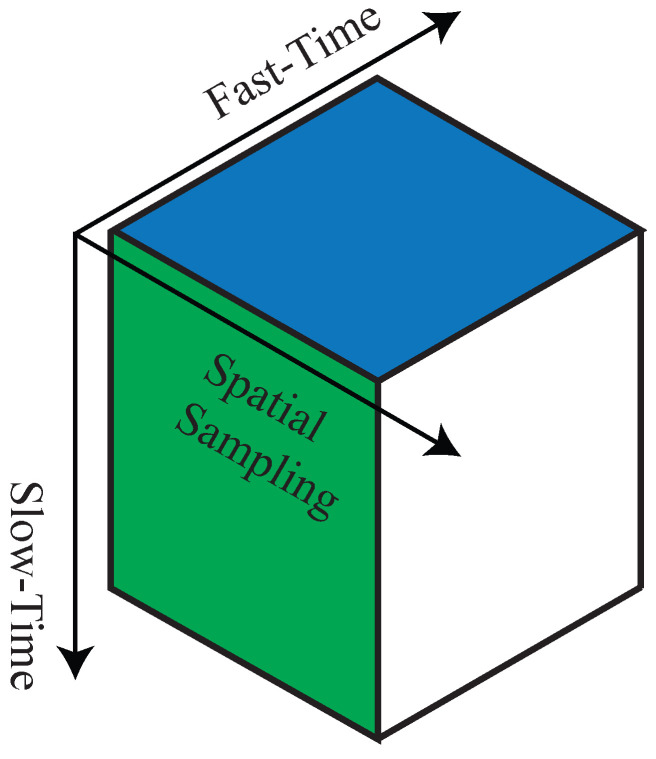
Datacube organisation of the complex samples. The green surface is the Slow-Time Spatial Sampling map, the blue one is the Fast-Time Spatial sampling map, and the white one is the Fast-Time Slow-Time map.

The complex samples of the beat signals are organised as a datacube: one axis is reserved for the samples of single chirps, another axis hosts the samples of different chirps, while the last hosts the samples of different virtual antenna elements. These axes are called Fast-Time, Slow-Time and spatial sampling, respectively. Figure 6 graphically represents the complex samples’ organisation.

**Figure 7:**
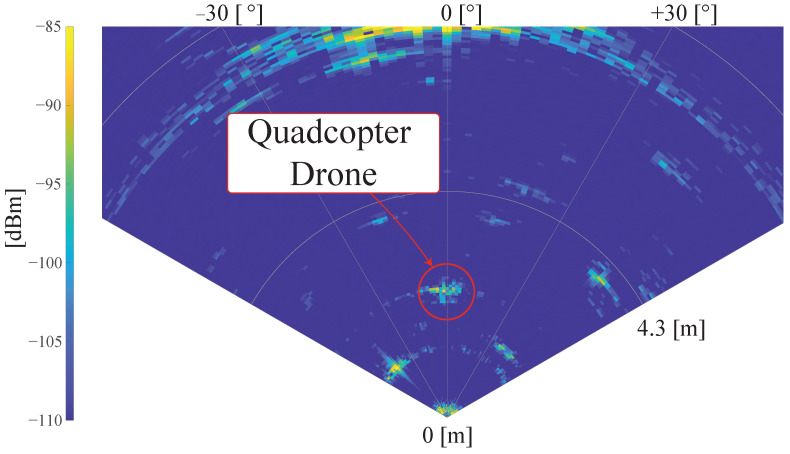
Example of radar Range-Angle detection map.

Converting the complex datacubes in detection maps requires a bi-dimensional Fast Fourier Transform (FFT) along the different faces of the datacube. Following the transformation, the three axes become the Range axis, the Doppler axis and the Angular axis, respectively. An example of a Range-Angle map is reported in Figure 7.

The applied technique used to extract the displacement vibration information from the radar signal is based on the identification of the propeller position on the Range-Angle map [24]. Once the desired “pixel” on the map is selected, it is possible to extract a complex vector along the Slow-Time axis, the phase of which encodes the displacement information. Indicating with *T* the Slow-Time axis, the phase signal can be written as

(2) $$\psi_{b}\left(nT\right)=\frac{4\pi f_{c}\;R_{0}+4\pi f_{c}\;x\left(nT\right)}{c},$$

where $R_{0}$ is the target distance, $f_{c}$ the carrier frequency, *c* the speed of light and x(nT) the displacement signal. Reverting Equation (2) allows us to extract the value of the vibration displacement x(nT). The exploited radar works in time division multiplexing (TDM) mode, meaning that the MIMO is implemented by using consecutive not overlapped transmissions of the transmitters. However, this operating mode limits the sampling frequency of the displacement signal. The value of *T* can be calculated as

(3) $$T=N_{tx}\;\cdot \;t_{chirp},$$

where $N_{tx}$ is the number of transmitters and $t_{chirp}$ the chirp duration. For brevity, Table 1 reports the values of the configuration parameters exploited in this work.

The method applied to measure the vibration displacement is the same as described in [24], which exploits the discrete Fourier transform (DFT) to calculate the frequency and amplitude of the vibration. In the cited work, the proposed method reports a mean displacement measurement error $\bar{\Delta D}=3.8\;\mu m\;$ with a standard deviation $\sigma_{\bar{\Delta D}}=11.6\;\mu m\;$ for a target angle $\theta_{r}$ = 0° placed at a distance $R_{0}=3.75\;m$. The error in reconstructing the temporal evolution of the vibration waveform is estimated with Root Mean Square Error (RMSE) in a timespan of 100 ms. It has a mean value $\bar{RMSE}=472.4\;\mu m\;$ and a standard deviation $\sigma_{\bar{RMSE}}=0.4\;\mu m$. Due to the dependence of the technique performance on the target range distance, it is possible to state that with a target positioned within an $R_{0}<3.75\;m$, such performance can be achieved also in the specific case under consideration in this study. In the next section the obtained radar vibration measurements will be described in more depth.

## 4. Dataset Description

**Figure 8:**
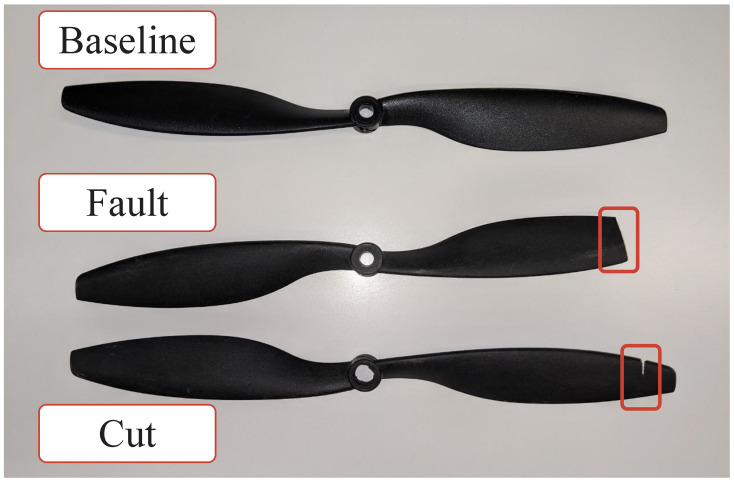
Propeller used to simulate the *Baseline* case, the *Fault* and the *Cut* classes.

The audio and vibration signals are collected from the drone, simulating different damages to one of its four propellers. In general, a *damage* to a propeller includes not only a change in its profile caused, for example, by accident or debris collisions, but also a variation in the aerodynamic profile of the propeller, due to wear [33]. As the latter class is difficult to simulate, ensuring repeatability in a laboratory environment, only the former is considered and, in the setup under test, the damaged propeller is always in the same position on the quadcopter chassis. Two different classes of damage are simulated, one called *Cut* and one called *Fault*, along with the *Baseline* class accounting for the intact propeller. The three classes are shown in Figure 8.

To gather a dataset as descriptive as possible, the propellers are modified on purpose to simulate the damages they usually undergo during flights. In the case of the *Fault* class, one tip of the propeller is completely truncated. This operation is performed by removing a portion of 0.5cmin one case, and 1cmin another. For the *Cut* damage class, the propeller is engraved at different distances from the tip. These are 0.5cm,1cm,1.5cmand 2cm. The set of tested cases can seem unbalanced, but simulating, for instance, a *Fault* of 1.5cmor 2cmdoes not have a practical sense, as the damage would be too severe to allow for any flight. The simulated failure classes were chosen based on the most common ones documented in the literature, considering that an overly severe failure would prevent the drone from flying correctly [34,35].

### 4.1. Audio Datasets

In order to ensure that the acquired audio measurements refer to the same experimental setup conditions, two audio datasets are collected simultaneously by exploiting in parallel both audio acquisition systems described in Section 3.2 (i.e., the professional and the embedded ones). For each condition of the propeller (i.e., *Baseline*, *Fault*, and *Cut*), recordings are carried out for 116 s, over three repetitions.

The audio dataset acquired by the professional microphones is composed of audio signals captured by three microphones placed around the quadcopter, as shown in Figure 2 and Figure 3. The initial sampling frequency of 48kHzused by the microphones is decimated by a factor of 3, obtaining a final sampling frequency of 16kHz. For each test, data composed of 1,856,000 × 3 × 3 samples are provided, where 1,856,000 is the number of samples in each audio signal, 3 is the number of repetitions, and 3 is the number of microphones. The audio signals are divided into subset windows of 4scomposed of 64,000 samples. This division provides a dataset of 64,000 × 29 × 3 × 3 samples for each test.

The dataset collected with the embedded audio acquisition system located onboard the drone is acquired at 16kHzsample rate, 16 bit per sample, and 4 s-long audio files.

### 4.2. Radar Vibration Displacement Signals

**Figure 9:**
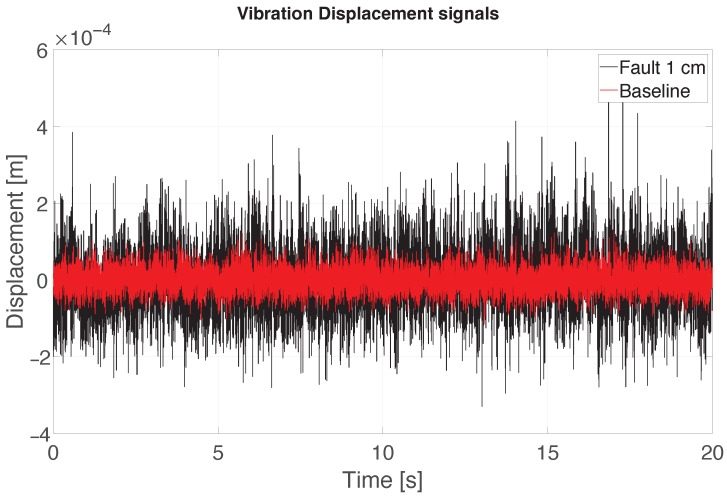
Example of 20sof vibration displacement signal in the cases of *Baseline* and *Fault* of 1cm.

The radar vibration signals are obtained after the processing stage described in Section 3.3. Such processing is applied to the beat signal samples collected during the different tests, resulting in a massive amount of data due to the configuration parameters chosen (e.g., an acquisition of 20simplies a raw data file of 7GB). Owing to this, collecting the beat signals for a long time is impractical because of the computational burden. For this reason, the radar acquisition is limited to 20.48sand repeated four times. Since the sensor fusion between audio and radar techniques takes place at a later stage, no synchronisation between the two acquisition systems is needed. An example of two acquired vibration displacement signals is reported in Figure 9.

With the radar configuration used, the vibration signal is sampled at 500 Hz. So, for each type of propeller (damaged and not), four vibration signals are collected, each one composed of 10,240 samples. On these signals, it is possible to apply the DFT to obtain both vibration displacement and frequency.

## 5. Machine Learning Model

In this section, the process leading to the creation of an ML model based on Neural Network (NN) and deployed to run on embedded platforms is described. The overall model design, and its hyperparameters tuning, were performed by a standard grid-search approach, exploring several feature selection processes and ML architectures.

### 5.1. Feature Extraction Phase

Inferences are performed on time series data output by microphones and processed by a sliding temporal window of 500ms, and a sliding step of 500ms(meaning that no overlapping occurs) is selected. Then, each of such temporal windows is preprocessed by Mel-scaled spectrograms, because of their wide employment and proven effectiveness for non-voice audio data [36,37,38]. Mel-spectrograms perform standard spectrograms from audio signals, and then they are filtered exploiting Mel-filterbanks, whose analytical formulation and definition can be retrieved in a previous work [39]. In this specific case, each frame forming the Mel-spectrograms is 20ms-long and overlapping by 10mswith the consecutive one, thus obtaining a 50% superimposition.

**Figure 10:**
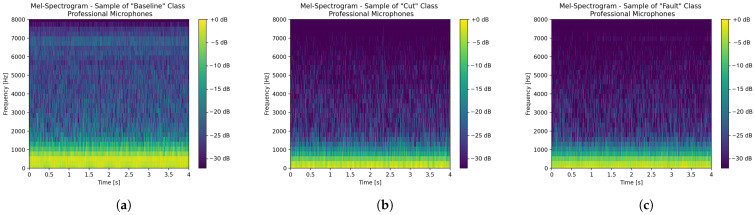
Mel-spectrogram samples, related to the acoustic measurements performed with the professional microphone-based data acquisition system, stemming from the preprocessing phase of the ML model: (**a**) *Baseline* class, (**b**) *Cut* class and (**c**) *Fault* class.

**Figure 11:**
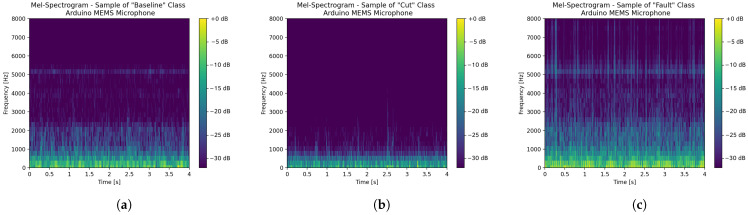
Mel-spectrogram samples, related to the acoustic measurements performed with the embedded data acquisition system (i.e., with the Arduino MEMS microphone), stemming from the preprocessing phase of the ML model: (**a**) *Baseline* class, (**b**) *Cut* class and (**c**) *Fault* class.

In compliance with the microphone sampling frequency of 16kHz, each frame contains 320 samples turning into the necessity of exploiting a 512 points FFT. Then, the spectrograms are filtered through 32 Mel-filterbanks, starting from 0Hz, meaning that the continuous component is preserved. Finally, the threshold of −32dB is selected as the noise floor, guaranteeing that no information related to sound is discarded. Figure 10 and Figure 11 respectively show the Mel-spectrograms of samples belonging to the three classes, in turn extracted from acquisitions performed with the professional microphone-based data acquisition system, and with the MEMS embedded data acquisition system.

Figure 10 and Figure 11 have a mere qualitative purpose because they show the Mel-scaled spectrograms associated with just three acquisitions. Indeed, showing such spectrograms for all of the acquisitions forming the datasets (i.e., 668 audio files including both audio datasets) is unfeasible herein. Nonetheless, some remarks can be drawn anyway, since the bulk of the spectrograms share common features. Specifically, for what concerns the acoustic measurements acquired with the professional microphones, spectrograms belonging to the *Cut* class (i.e., Figure 10b) and those belonging to the *Fault* class (i.e., Figure 10c) are quite similar, since the types of fault and cut simulated are pretty mild (see Figure 8) and do not significantly change the drone sound emission. On the other hand, the spectrograms belonging to the *Baseline* class (see Figure 10a) have a power that is more spread over the considered spectrum. Generally, all the spectrograms share a high informative content at low frequencies (i.e., less than 1 kHz). These are surely related to the noise coming from the motors, rather than the one generated by the propellers. However, since the motors are no longer stabilised whenever propellers are damaged, they tend to vary the noise related to their spinning, and this is the reason why the continuous component is preserved. On the other hand, spectrograms belonging to the *Cut* and *Baseline* classes are characterised by a power spectral information which is prevalent below 6 kHz, and barely absent above such a threshold. Conversely, spectrograms belonging to the *Baseline* class show a marked informative content up to 7 kHz. Regarding measurements performed with the embedded acquisition system, the relative spectrograms are rather different from those of Figure 10. The main reason is that the exploited sensor is a MEMS microphone, which is also capable of sensing the vibrations propagating along the drone chassis whenever it is mechanically integral installed to it (as in this case), together with the proper acoustic noise generated by the propellers. This also translates into two peculiar features: the power distribution at low frequencies is more concentrated (i.e., less than 500 Hz) with respect to that of the spectrograms of Figure 10; in the spectrograms belonging to *Baseline* and *Fault* classes a clear spectral power component at 5 kHz can be seen, which is reasonably attributed to the mechanical vibrations propagating through the drone chassis, and that is less evident for spectrograms belonging to the *Cut* class, because such an instance of damage alters the mechanical properties of the drone, modifying the relative vibrations. Additionally, the spectral power associated with the spectrograms of Figure 11 is generally less spread than those of Figure 10, because the embedded MEMS microphone has a poorer dynamic with respect to that of the professional equipment.

### 5.2. Neural Network

A two-dimensional Convolutional Neural Network (CNN), taking as input the Mel-scaled spectrograms, is selected for audio classification, which is developed by making use of TensorFlow and Keras Python modules. The NN is composed of three stacked convolutional layers accounting for 16 neurons each. Each layer of the network has a pooling layer that computes the maximum value from the previous convolutional layer. This maximum value is then stacked on top of the previous layer. This is done in order to perform a nonlinear down-sampling, so to achieve a reduced model complexity, translating into a lesser required computational power, that is more suitable to be run on a microcontroller. All of the CNN neurons have Rectified Linear Unit (ReLU) as activation function. ReLU is still a nonlinear function that can be easily implemented by resorting to an if/else statement. Finally, a flattening layer is exploited to convey data to a dropout layer, having a rate 0.25, and a softmax layer having three outputs (i.e., as many as the target classes) providing the output classes probabilities related to the input sample at hand.

The network is trained twice: firstly over the dataset composed of the acoustic measurements collected with the professional microphone acquisition system, and secondly over the dataset formed by the measurements accomplished with the embedded data acquisition system. Then, training is performed in both cases by standard training-validation method exploiting the standard backpropagation algorithm, and by considering the categorical cross entropy as a loss function [39]. To this end, the datasets introduced in Section 4.1 are split into training, validation, and test sets with a ratio of 0.6, 0.2, and 0.2, respectively.

### 5.3. Embedded Machine Learning Model

The ML model is converted into its tinier version, to be deployed on the nRF52840 microcontroller board, by making use of the TensorFlow Lite framework, which allows us to convert the ML model into a C++ library to be included in the microcontroller firmware, compiled and deployed on the device. Then, two versions of the converted ML model are obtained, differing in the type of variables they account for the NN weights. In particular, the complete version uses 32bit floating point variables, and the quantised one uses 8bit integer variables. This is done to trade off model performance and memory size, since the complete version is usually finer and requires more hardware resources, while the quantised one is typically coarser, but lighter.

## 6. Results and Discussion

### 6.1. Embedded ML Audio Model Classification Performances

**Figure 12:**
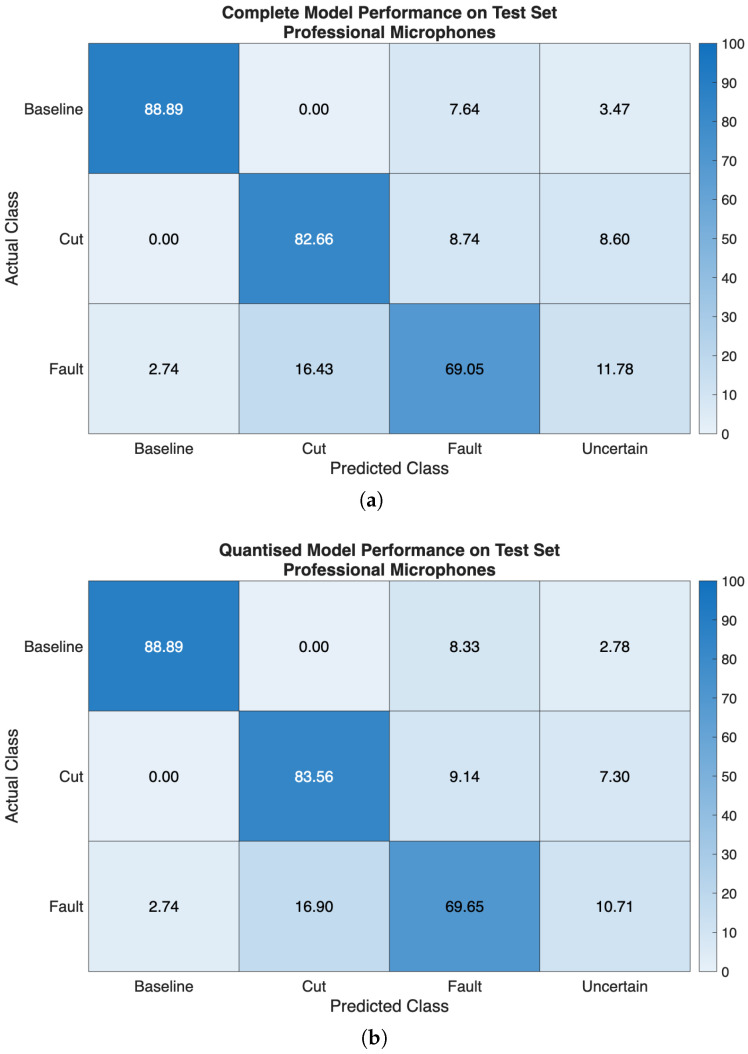
Classification probabilities, in percentage, showing classification performance on the test set extracted from the dataset gathered with the professional microphones: (**a**) complete model exploiting 32-bit floating point variables, and (**b**) quantised model exploiting 8-bit integer variables.

**Figure 13:**
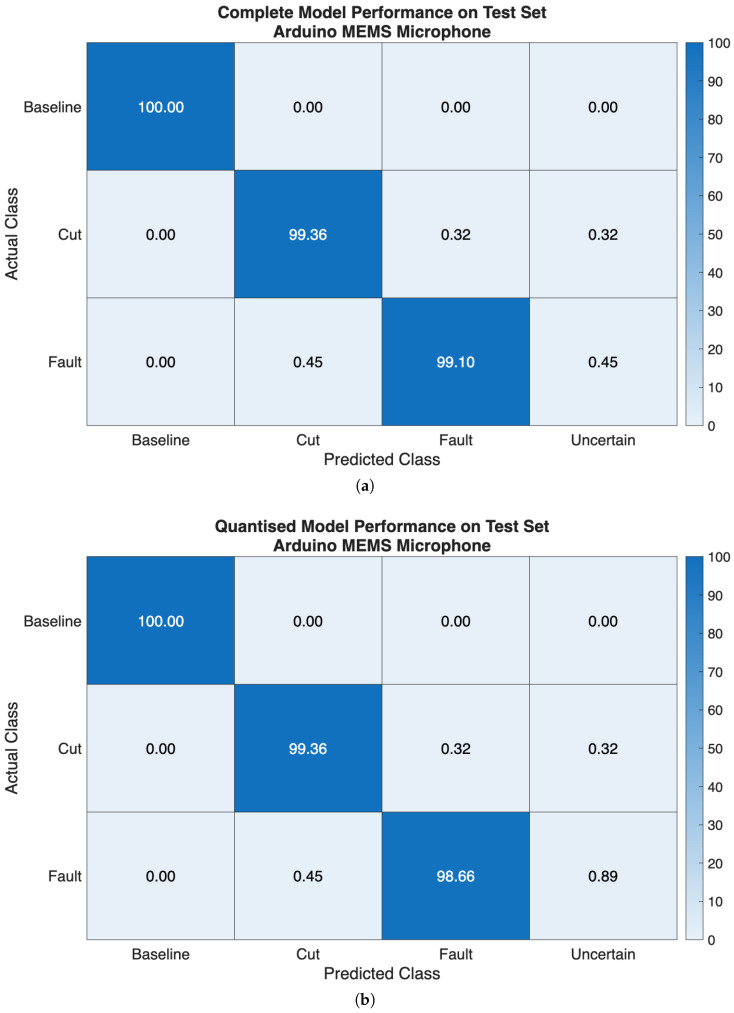
Classification probabilities, in percentage, showing classification performance on the test set extracted from the dataset gathered with the embedded Arduino MEMS microphone: (**a**) complete model exploiting 32-bit floating point variables, and (**b**) quantised model exploiting 8-bit integer variables.

The performance of the ML model in terms of classification probabilities, in percentage, on the test set extracted from both audio datasets, is shown as classification probabilities, in percentage, in Figure 12 and Figure 13.

When the model is trained and tested over the dataset obtained from the professional microphones, almost equal performances are seen on the test set, regardless of the considered variants. Indeed, the relative mean classification performance are 80.20% for the complete model, and 80.70% for the quantised model on the test set. Samples belonging to the *Baseline* class were correctly identified 88.89% of the times, respectively, for both model versions. Then, the samples belonging to the *Cut* class were identified 82.66% and 83.56% of the times, in turn, by both complete and quantised models. On the other hand, examples forming the *Fault* class experienced poorer classification rates, 69.05% and 69.65% of the times in turn, since misclassifications occurred in favour of the *Cut*. This result could be a consequence of the strong similarity of the spectrograms related to such classes (see Figure 10b,c). However, it can also be remarked that such a model can clearly distinguish between *Baseline* examples and input data affected by a damage instance (i.e., *Cut* and *Fault*), regardless of the specific type. In Figure 12 and Figure 13, a fourth class named *Uncertain* is also displayed. This class represents cases where the output class probabilities of the input sample are within the range [0.4,0.6]. Consequently, the classification result is considered uncertain because no single class has a predominant probability. This approach is based on a conservative assumption, where thresholds are arbitrarily chosen based on previous experience. In practical terms, relying on a prediction with a probability close to 0.5 can be misleading and potentially disruptive, such as suspending a drone flight due to a false failure or failing to take countermeasures to a failure that was not identified. However, this approach has a significant impact during operational flights, because it enables more informed decision-making. Additionally, it is reasonable to expect that a prediction labelled as *Uncertain* will change over time and fall into one of the other considered classes. This is especially true if the potential failure cause becomes more severe and easier to detect during the flight.

Conversely, the model performs significantly better when trained over the dataset containing the measurements acquired with the embedded microphone, regardless of the considered variants. Specifically, on the test set, a mean classification performance of 99.49% and of 99.34% is obtained, for the complete and quantised versions of the model in turn. This enhancement in performance can be due to the fact that the MEMS microphone senses also the vibrations propagating through the drone chassis, along with the acoustic noise coming from the propeller, in contrast to audio professional microphones. This further justifies the inclusion of the onboard microphone to achieve fault detection, with clear advantages with respect to the professional microphone setup.

**Figure 14:**
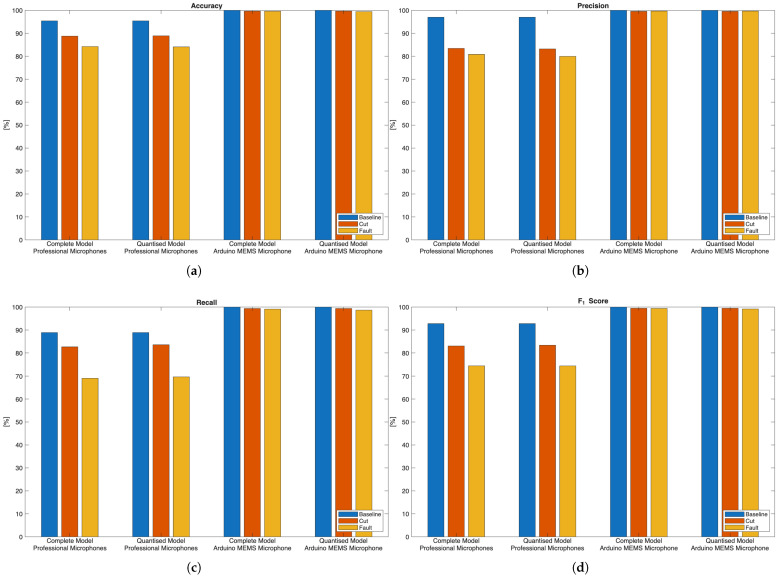
Performance comparison of the developed embedded ML models over the following metrics: (**a**) accuracy, (**b**) precision, (**c**) recall and (**d**) $F_{1}$-score.

According to such results, the first stage of the non-contact sensors fusion paradigm we are proposing can be satisfactory accomplished by the embedded ML model, that is trained over the dataset acquired with the MEMS microphone, running on the onboard microcontroller platform, since it is capable of a real-time continuous detection of the occurrence of damage to the propeller. Indeed, if the classes *Cut* and *Fault* are joined together in a metaclass *Damage*, then the model is capable of detecting such an event in almost all the cases, regardless of the model version. Then, by resorting to the radar-based contactless analysis, further information regarding the type of damage that occurred can be retrieved, according to a sensor fusion approach. Finally, both the model versions can be further analysed by comparing their performance over the accuracy, precision, recall and $F_{1}$-score metrics, as shown in Figure 14. This gives additional insight, but still remarking that the models trained and tested over the dataset collected by the onboard MEMS microphone outperform their counterparts relying on the dataset acquired with the professional microphones.

Both model versions, regardless of the type of dataset exploited for the training and testing phases, are compared from the point of view of the hardware requirements in Table 2. Owing to the types of variables it exploits, the quantised version extremely optimizes the hardware requirements. In particular, it needs 41.9kBof RAM and 56.4kBof flash memory. On the other hand, the complete version respectively needs of 152.1kBand 72.6kBof RAM and flash memory. In other words, due to the comparable classification performances of the model versions, the quantised one is by far preferable, because it allows more room for additional routines that the detection system can implement. In addition, the quantised version can be executed by the microcontroller faster than the complete one (i.e., 486.0msand 2200.0msin turn), meaning that it is better suited for the real-time detection application.

### 6.2. Radar Displacement Results

The obtained vibration displacement signals are sampled with a lower sampling frequency than the audio signals. Due to this, longer temporal windows are needed to obtain information about the damage applied to the propeller. The signal samples are then divided in windows of 2.048 s where each one contains 1024 samples. It is possible to carry out a statistical analysis based on the mean values of vibration frequency and displacement measured by exploiting a DFT-based approach. The noisy nature of the vibration itself, and the low signal-to-noise ratio (SNR) of the radar echo, make the application of a filtering process necessary. This process is performed in two steps: the former is the application of the Savitzky–Golay filter, followed by a high-pass filtering useful to remove the low-frequency components [40,41]. The Savitzky–Golay filter impulse response is dependent on two different parameters: the filter polynomial order, and the frame length. These parameters are indicated with $SG_{order}$ and $SG_{length}$, respectively. The filtering procedure is applied to the 1024 samples vibration signal segments. After the filtering, the DFT can be computed. The parameters of the filters are summarised in Table 3.

The rationale behind choosing the values of the parameters of the Savitzky–Golay and high-pass filters is as follows:

- $SG_{order}$ and $SG_{length}$ are chosen according to the vibration peak frequency. With $SG_{order}=3$ and $SG_{length}=9$, the first zero of the magnitude frequency response of the filter is 98.14 Hz, while the peak of the vibration is 49.31 Hz. This way, its frequency falls within the first lobe of the filter.
- The high-pass filter is used to remove the low vibration frequencies. The method used to obtain the displacement measurement is to select the highest peak and store the collected value. As the low frequency components can be high but not significant for the proposed method, the high-pass filter is used to remove them.

**Figure 15:**
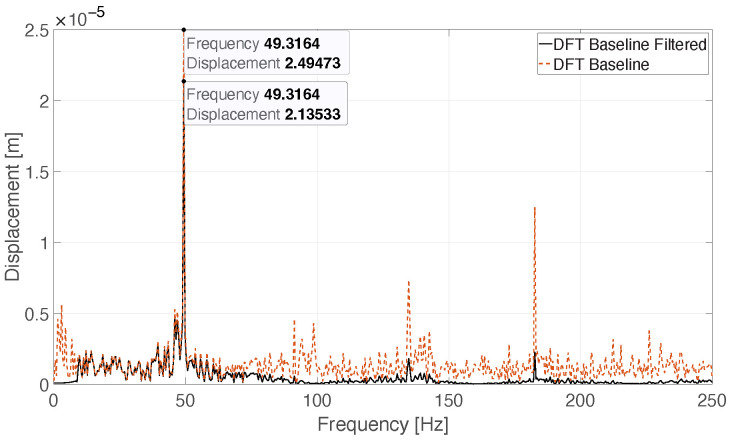
Comparison between the DFT of a *Baseline* displacement signal and its filtered version, respectively, the black line and the red dotted one.

The effect of the filtering process, applied to a sample of the *Baseline* signal, is illustrated in Figure 15.

From all the computed DFTs, the main peak value is extracted, and the relative frequency and displacement amplitude are stored. Forty values for each type of propeller are obtained, and the mean values and standard deviations of the frequencies and displacements are calculated. Let us reference to the mean values and standard deviations respectively as ${\bar{q}}_{f}$ and $\sigma_{{\bar{q}}_{f}}$ for the vibration frequency, and, in turn, as ${\bar{q}}_{D}$ and $\sigma_{{\bar{q}}_{D}}$ for the vibration displacement amplitude. Therefore, the obtained values are reported in Table 4.

**Figure 16:**
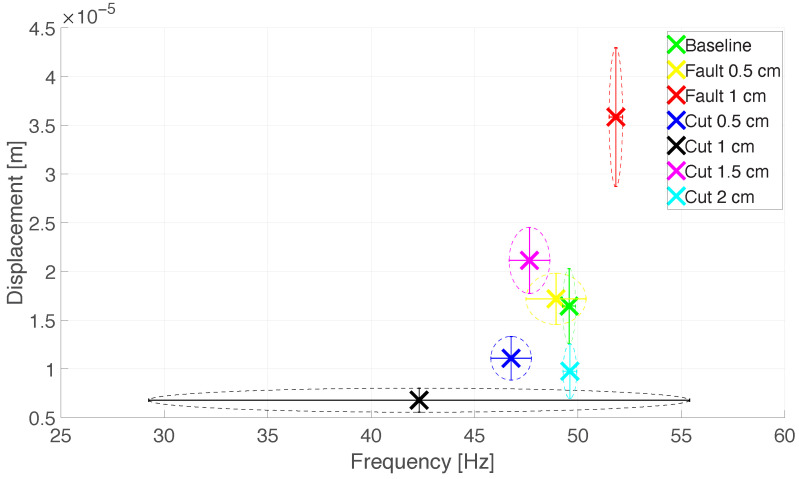
Scatter plot of the mean values and standard deviations of displacement frequency and amplitude. The dotted lines indicate the different damage area regions.

The obtained results can be represented in a scatter plot, reporting on the abscissa the frequency values of ${\bar{q}}_{f}$ and $\sigma_{{\bar{q}}_{f}}$, while on the ordinate the values of ${\bar{q}}_{D}$ and $\sigma_{{\bar{q}}_{D}}$. This representation is reported in Figure 16.

Figure 16 shows that the mean values can be used to retrieve the Cartesian coordinates of the centre of a specific region, indicated with the coloured cross. The standard deviations can be exploited to define the limits of the regions and to provide an indication of their extent. Such regions are in some cases not overlapping, thus facilitating their identification. Indeed, the cases of *Baseline*, *Fault* 1 cm,*Cut*0.5 cm,*Cut* 1 cm,*Cut*1.5 cmand *Cut* 2 cmcan be easily separated and promptly identified. There is a partial overlapping between the regions of the *Baseline* and *Fault* 2 cmcases. However, this condition is reasonable since such a mild fault implies a negligible modification of the propeller, that highly resembles to an intact one. Moreover, a propeller damaged that way would shortly experience a more severe fault (e.g., a cut of the faulted tip), thus becoming much easier to detect by the proposed analysis approach. In general, the results demonstrate the ability to effectively refine the identification of the type of damage on the propeller, by the joint use of the radar sensor and the proposed technique.

### 6.3. Discussion

It is possible to start the discussion of the results from the obtained performance of the audio measurements classification. This step is designed to provide an early real-time detection of the type of damage the propeller underwent. The matrices of the complete and quantised models, when trained and tested over the two collected datasets, demonstrate how the developed method can reveal the type of damage with good performances, when it relies on the acoustic measurements acquired by exploiting the MEMS microphone. Moreover, the proposed approach proves to be more effective with respect to that of the previous work [7], which only relied on the acoustic measurements acquired with the far located professional microphones. Specifically, if considering the mean classification performances of the complete model, an improvement of 9.09% is now achieved, thus achieving 99.49% performance; conversely, for the quantised model, such an enhancement reaches 9.88%, thus obtaining 99.34% performance. In addition, the proposed methodology for the considered application scenario makes sense because it suits well to be physically deployed onboard drones, and it is capable of making predictions in a limited timespan, especially if the quantised model is used (as confirmed by the results reported in Table 2). Therefore, at this point, the proposed system can benefit from the support of the closest ground radar station, which receives the preliminary fault prediction of the embedded ML model onboard the drone, and starts to analyse the drone vibration signal.

The radar results demonstrate how the single classes related to an instance of damage can be distinguished by evaluating the vibration displacement measurements. To obtain satisfying results, the DFT computation is performed on 1024 samples of the vibration signal. From these values, the vibration displacement amplitude and the frequency can be extracted: when considered jointly, these quantities locate specific regions on the displacement-frequency plane, depending on the specific propeller condition they relate to. The calculated standard deviations define an area around the mean values, and, by locating the position of the displacement-frequency measurement in the graph illustrated in Figure 16, it is possible to determine the corresponding class of damage. Comparing the audio measurements classification and the radar measurements method, the latter can better identify different regions for the *Cut* class. The regions for this type of damage are more distinguishable than the one of the *Fault* case.

Figure 16 also shows that the *Baseline* and the *Fault* 0.5 cmidentify regions with a heavy overlap, for which also the mean values of displacement amplitude and frequency are close together. In contrast, the *Cut* damage classes correspond to regions in the plane that can be clearly distinguished. This way, the radar method makes it possible to integrate the preliminary information provided by the audio signal classification. Indeed, the radar can extract more in-depth information, and through the capability of identifying the position of the damaged propeller it can also indicate which one of the four installed on the chassis is damaged. The combination of both detection approaches realizes a diagnostic system that is both time-responsive and accurate in defining the type, the amount, and—for the *Cut* class—the location, of the damage. Therefore, a two-step non-contact sensor fusion technique is carried out, thus satisfying the requirements of the considered application scenario, as they were set in Section 2.

The presented results are obtained within conditions that are functional to demonstrate the feasibility of the two-step methodology in a controlled fashion. External environmental effects, like wind or outdoor background sounds, are intentionally excluded from the analysis, striving to achieve a deterministic setup. For example, wind will affect the control system of the drone, which will compensate such an effect by tuning the PWM control signals driving the propellers. Therefore, removing such confounding conditions from the investigation makes the presented results reliable and repeatable.

## 7. Conclusions

The operational safety of UAVs requires the development of fault detection systems capable of spotting and identifying the type and severity of a fault in the shortest time possible, so as to enable effective countermeasures. This work presented a two-step sensor fusion methodology aimed at assessing the damage to UAV propellers, by means of an onboard system exploiting a tiny ML classifier trained on traceable acoustic measurements, for a first real-time (or near real-time) detection and coarse classification of the damage. Based on the outcome of the classification process, that has been proved with a classification performance higher than 99%, a second step is possibly applied, in which a ground-based radar system performs vibration displacement and frequency measurements on the affected UAV, to quantify the severity of the propeller damage and eventually opt for the forced landing of the drone. Future works will address a greater variety of possible damage and faults, to further extend the generalisation of the methodology and increase the robustness of the proposed approach. This work demonstrated a first validation of the proposed two-step methodology. The performed tests confirm the capability to reveal propeller damages with the combination of audio and radar measurements. Nonetheless, in a more realistic condition, wind and background noise will affect the audio measurements. To make the method reliable against such phenomena, it must include techniques able to cope with wind and background noise, achieving their suppression. However, at the same time further sensors could be included, thus enriching the sensor fusion paradigm. For instance, accelerometers are robust towards wind and acoustic noise by default, meaning that their measurements can potentially improve the proposed system, thus augmenting the audio-based methodology. Prior to this stage, the effects of the disturbances on the audio-based method should be studied, and a countermeasure suited for embedded implementation should be developed. This can be either, or both, hardware- or algorithmic-based. For instance, in [26] some tape is placed on the microphones to reduce wind effect, but this causes the sensor to behave more like an accelerometer [28] rather than like a proper microphone. Signal processing-based approaches, like the ones proposed in [42,43], need to be suitable for embedded implementation and inference time. Both approaches have pros and cons to be evaluated in outdoor implementations. Then, the next development steps will involve outdoor measurements with external phenomena investigation and quantification.

## Figures and Tables

**Table 1:** Radar parameters.

Parameter Name	Value
$f_{start}$	77 GHz
*B*	3.99 GHz
$t_{chirp}$	167 µs
$N_{tx}$	12
*T*	2 ms
$n_{samples}$	512
$f_{s}$	12 MHz

**Table 2:** Hardware performances comparison between the two versions (i.e., complete and quantised) of the model. Notice that the deployment platform accounts for 256kBof RAM, and 1MBof flash memory.

Model Version	RAM Occupancy	Flash Occupancy	Execution Time
Complete	152.1 kB	72.6 kB	2200.0 ms
Quantised	41.9 kB	56.4 kB	486.0 ms

**Table 3:** Filtering process parameters.

Filter Type	Parameter Name	Value
Savitzky–Golay	$SG_{order}$	3
Savitzky–Golay	$SG_{length}$	9
High Pass	Type	Butterworth
High Pass	Pass Frequency	10 Hz
High Pass	Filter Order	3

**Table 4:** Results of the statistical analysis performed on the radar signals.

Propeller Status	${\bar{q}}_{f}$ [Hz]	$\sigma_{{\bar{q}}_{f}}$ [Hz]	${\bar{q}}_{D}$ [μm]	$\sigma_{{\bar{q}}_{D}}$ [μm]
*Baseline*	49.57	0.31	16	4
*Fault* 0.5 cm	48.94	1.45	17	3
*Fault* 1 cm	51.83	0.32	36	7
*Cut* 0.5 cm	46.76	0.98	11	2
*Cut* 1 cm	42.32	13.07	7	1
*Cut* 1.5 cm	47.66	0.99	2	3
*Cut* 2 cm	49.61	0.32	10	3

## Data Availability

The datasets presented in this article are not readily available because of technical and ethical limitations. Requests to access to the datasets should be directed to the authors.
